# Neutral Processes Drive Seasonal Assembly of the Skin Mycobiome

**DOI:** 10.1128/mSystems.00004-19

**Published:** 2019-03-26

**Authors:** Xinzhao Tong, Marcus H. Y. Leung, David Wilkins, Hedwig H. L. Cheung, Patrick K. H. Lee

**Affiliations:** a School of Energy and Environment, City University of Hong Kong, Hong Kong SAR, China; University of Waterloo

**Keywords:** community assembly, human skin, skin mycobiome, temporal dynamics

## Abstract

The importance of microorganisms to human skin health has led to a growing interest in the temporal stability of skin microbiota. Here we investigated the dynamics and assembly of skin fungal communities (mycobiomes) with amplicon sequencing of samples collected from multiple sites on 24 healthy Chinese individuals across four seasons (in the order of winter, spring, summer, and autumn in a calendar year). We found a significant difference in community compositions between individuals, and intrapersonal community variation increased over time at all body sites. Within each season, the frequency of occurrence of most operational taxonomic units (OTUs) was well fitted by a neutral model, highlighting the importance of stochastic forces such as passive dispersal and ecological drift in skin community assembly. Despite the significant richness contributed by neutrally distributed OTUs, skin coassociation networks were dominated by taxa well-adapted to multiple body sites (forehead, forearm, and palm), although hub species were disproportionately rare. Taken together, these results suggest that while skin mycobiome assembly is a predominantly neutral process, taxa that could be under the influence of selective forces (e.g., host selection) are potentially key to the structure of a community network.

**IMPORTANCE** Fungi are well recognized members of the human skin microbiota and are crucial to cutaneous health. Common cutaneous diseases such as seborrheic dermatitis and dermatophytes are linked to fungal species. Most studies related to skin microbial community dynamics have focused on Western subjects, while non-Western individuals are understudied. In this study, we explore the seasonal changes of the skin mycobiome in a healthy Chinese cohort and identify ecological processes that could possibly give rise to such variations. Our work reveals the dynamic nature of host skin fungal community, highlighting the dominant roles neutral forces play in the seasonal assembly of skin mycobiome. This study provides insight into the microbial ecology of the human skin microbiome and fills a knowledge gap in the literature regarding the dynamics of skin fungal community.

## Abstract (precis)

Fungi are well recognized members of the human skin microbiota and are crucial to cutaneous health. Common cutaneous diseases such as seborrheic dermatitis and dermatophytes are linked to fungal species. Most studies related to skin microbial community dynamics have focused on Western subjects, while non-Western individuals are understudied. In this study, we explore the seasonal changes of the skin mycobiome in a healthy Chinese cohort and identify ecological processes that could possibly give rise to such variations. Our work reveals the dynamic nature of host skin fungal community, highlighting the dominant roles neutral forces play in the seasonal assembly of skin mycobiome. This study provides insight into the microbial ecology of the human skin microbiome and fills a knowledge gap in the literature regarding the dynamics of skin fungal community.

## INTRODUCTION

Skin is the major interface between the human body and its external environment (1). The microbial communities (microbiota) residing on skin, mostly consisting of bacteria and fungi, are important in protecting human hosts from pathogen invasion (2, 3). Temporal shifts in skin microbial composition may be associated with cutaneous fungal infections (4, 5), and the incidence and severity of skin diseases vary with seasonality and environmental fluctuations (6–8). Thus, understanding the determinants of skin microbial dynamics and how skin communities are assembled over time provides a framework for skin health prediction, risk assessment, and disease treatment.

Previous metagenomic and amplicon-based studies have characterized the skin microbiota across multiple body sites of Western subjects (9–11). Due to the physiological difference between skin niches (9), both bacterial and fungal communities display topographical distributions (10, 12): for example, with lipophilic taxa enriched particularly on sebaceous sites, while other taxa characterize dry and moist sites (13). In addition, foot sites are recognized to have the highest fungal diversity and exhibit a higher variability compared to other body sites (10, 11). A recent mycobiome study has shown that skin fungal diversity converges from childhood to adulthood, with a profound shift in community composition during puberty (14). In healthy adults, *Malassezia* species such as Malassezia restrica, Malassezia globosa, and Malassezia sympodialis have been identified as core members of the skin mycobiome, which are largely stable over time (11, 15), while a decrease in *Malassezia* fungal diversity could be associated with skin diseases (16) or therapeutic treatment (17). In addition to host-intrinsic factors, stochastic forces like the transient acquisition of microbes by dispersal from external environments (18), microbial transmission between cohabiting members of a residence (19–21), and ecological drift (22) may also affect the diversity and composition of skin microbiota.

As a result, these host-associated microbial communities are typically uneven, with few abundant taxa and many rare taxa, reflected in a long-tail rank abundance distribution (23). To date, our knowledge of many ecosystems, including skin, is mostly based on the dominant taxa, but rare species are receiving increased attention as they may have a disproportionate influence on community stability and function (24–27). Taxa that are usually rare but occasionally become abundant, namely the conditionally rare taxa (CRT) (26, 28), are of particular interest. In drinking water distribution systems, nitrifiers bloom to abundance after disturbance, and these CRT have been suggested to be indicators of environmental changes that are otherwise difficult to detect (29). The rare-to-abundant occurrence pattern of CRT has been reported in multiple ecosystems, where CRT ecology explains large temporal shifts in the structure of microbial communities (25, 26) and may help to identify the biological, chemical, and physical drivers of microbial dynamics.

Given the various factors associated with community dynamics in the microbiomes of healthy individuals (30), there is growing interest in using theoretical models to query experimental data to identify processes that may drive such variations. Neutral theory is frequently applied to predict the assembly pattern of microbial communities and is favorable for its relative simplicity. This model assumes that all species in the community are functionally equivalent and that stochastic factors (i.e., random dispersal and birth/death events) are the primary drivers of ecological diversity and community structure (31). This approach has been successfully applied to model microbial communities in ecosystems, including the human lung (32, 33) and skin (34), animals (35, 36), and wastewater (37). On the other hand, a simpler approach is the binomial distribution model, which assesses the importance of random sampling on microbial community structure in the absence of drift and dispersal limitations (38), which is a useful complement to the neutral model for understanding microbial community assembly.

In this study, we investigated the dynamics of skin fungal communities of a healthy Chinese cohort across four seasons. Furthermore, we investigated the relative contributions of individual-specific CRT to the temporal dynamics of individual mycobiomes and whether there were signature taxa that uniquely identified hosts. Finally, we quantified the importance of neutral processes in skin community assembly and used coassociation networks to shed light on the potential interactions between fungi. Overall, this study provides important insights into the seasonal dynamics of the skin mycobiome in an Asian cohort and expands our understanding of how stochastic and deterministic processes drive the assembly of human-associated microbial communities. In particular, this study highlights the importance of seasonality in network stability of the skin mycobiome, with a more fragile network in autumn than other seasons. These findings can help us understand the links between seasonal variation of the skin mycobiome and the seasonality of certain skin disorders and can be useful for the prevention and treatment of cutaneous diseases.

## RESULTS

### Intrapersonal skin mycobiome composition varies over time.

**FIG 1:**
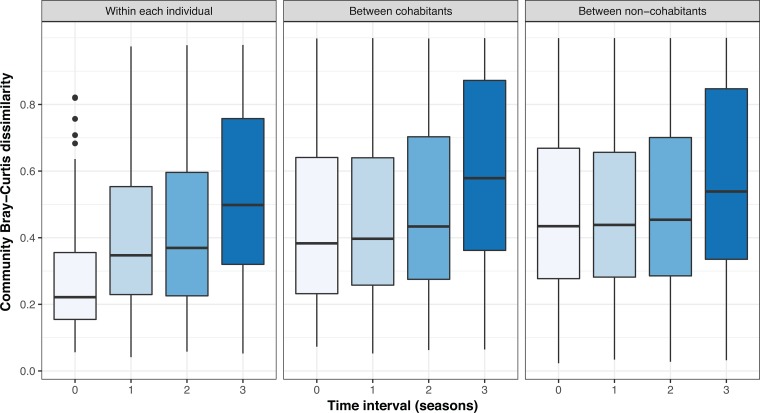
Effect of time on skin community dissimilarity. Community Bray-Curtis dissimilarity was calculated for the same anatomical site within and between individuals (*n *=* *24). The comparison groups include within each individual, between cohabitants, and between noncohabitants. Time interval is the number of seasons between sampling. The intrapersonal community dissimilarity at the time interval zero refers to the pairwise comparison between the symmetrical sites (left/right palms or forearms) within the same individual and season.

The temporal stability of skin mycobiomes was investigated on a per-individual, per-site basis using the Bray-Curtis dissimilarity metric. With symmetrical sites combined, intrapersonal community dissimilarity dramatically increased after one season, almost doubling after three seasons (Fig. 1), suggesting that the long-term intrapersonal skin community similarity was lower than the short-term one. This pattern held for all body sites, with the change in forehead community composition lowest among the three sites over the same time period (see Fig. S1A in the supplemental material), consistent with the relative stability of sebaceous sites compared to dry sites (11). When community dissimilarity between individuals was considered, cohabitants harbored more similar communities than noncohabitants, though this effect was no longer observed after an interval of three seasons (Fig. 1).

10.1128/mSystems.00004-19.1 FIG S1 Temporal stability of the skin community at different body sites. (A) Effect of time on intrapersonal community Bray-Curtis dissimilarity at different anatomical sites. The time interval is the number of seasons between sampling. (B) Correlation between intrapersonal community diversity and dissimilarity at different anatomical sites. Points represent forehead (*n* = 24), forearm (*n* = 48), and palm (*n* = 48) communities across four seasons. *P* values were calculated using the R function “cor.test.” Download FIG S1, PDF file, 2.5 MB. Copyright © 2019 Tong et al. 2019 Tong et al. This content is distributed under the terms of the Creative Commons Attribution 4.0 International license.

To examine whether intrapersonal community stability correlated with diversity, the correlation between the abundance-based Shannon diversity index and Bray-Curtis dissimilarity metric was tested. A positive rank correlation was observed for both palm and forearm communities (Spearman’s rho = 0.41 and 0.30, *P* = 0.003 and 0.04, respectively [Fig. S1B]), but this trend was insignificant in forehead communities (*P* = 0.19). In addition, a significant temporal change in diversity was detected for both palm and forearm sites (Kruskal-Wallis test, *P* = 3.3 × 10^−5^ and 1.6 × 10^−4^, respectively), but not for forehead (*P* = 0.78). These results suggest that skin community stability varies over time as a function of diversity for upper limbs, where low-diversity sites appear to be more stable than high-diversity sites.

### Intrapersonal skin mycobiome varies significantly at the OTU level across seasons.

Next, we assessed the temporal stability of dominant fungal taxa within each host community at the rank of genus. The skin commensal *Malassezia* remained largely stable over time as a core genus across all individuals (with *M. restricta* and *M. globose* dominating) (see Fig. S2 in the supplemental material), and forehead harbored a higher abundance of *Malassezia* than palm and forearm (Kruskal-Wallis test, *P* = 9.7 × 10^−8^). This finding suggests that sebaceous skin sites are more selective for members that have the ability to metabolize lipids present in the sebum compared to dry sites (39). Less dominant and ubiquitous genera varied widely in abundance at different body sites (Fig. S2). Specifically, taxa including *Aspergillus*, *Candida*, and an unclassified member within the order *Tremellales* tended to be enriched at specific sites of certain individuals, but could also be periodically absent, low in abundance, or fluctuate widely in other subjects.

10.1128/mSystems.00004-19.2 FIG S2 Temporal stability of the top 15 genera and the abundance profile of *Malassezia* species. (Left panel) Bubble heat maps are colored based on the mean relative abundance of a genus within a host body site, and bubble size is proportional to the CV of the relative abundance of a genus across seasons. The absence of a bubble means that genus was not detected at a host body site in any season. (Right panel) Bar charts show the mean relative abundance of *Malassezia* species at different body sites of individuals across seasons. Download FIG S2, PDF file, 1.5 MB. Copyright © 2019 Tong et al. 2019 Tong et al. This content is distributed under the terms of the Creative Commons Attribution 4.0 International license.

**FIG 2:**
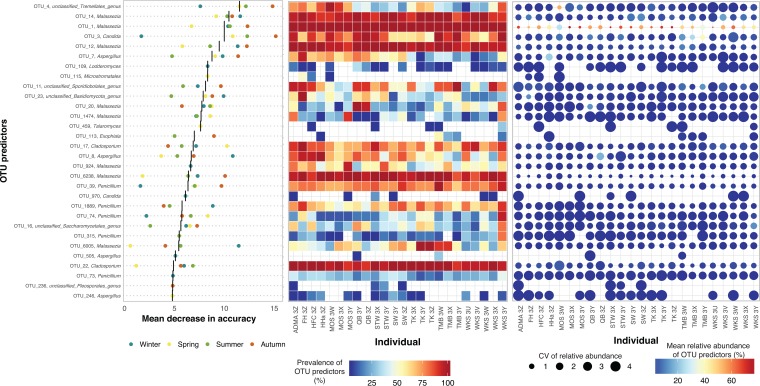
Discriminatory power and distribution pattern of OTU predictors across individuals and seasons. (Left panel) The top 30 OTU predictors are selected and ranked in decreasing order of the mean discriminatory power (black line, mean decrease in accuracy score across four seasons) based on the random forests algorithm, with taxonomy assigned at the genus rank (*y* axis). The mean decrease in accuracy score for each OTU predictor in each season is color-coded. (Middle panel) Prevalence (i.e., occurrence frequency) of OTU predictors in an individual community. (Right panel) Mean (color of nodes) and coefficient of variation (CV) (size of nodes) of the relative abundance of OTU predictors in an individual community. The absence of nodes in the middle and right panels indicates these OTU predictors were not detected in the host community at any season. The three panels are constructed and arranged following the format proposed by Oh et al. (11).

As individuals with unique microbial fingerprints (i.e., signature taxa) can be differentiated from one another at intervals spanning months or years (11), supervised random forest classifiers were used to study whether host-specific skin fungal signature taxa exist and persist across seasons. Although the resolving power of the top 30 OTU predictors varied over time, one signature taxon (OTU_4, within *Tremellales*; mean relative abundance of 54.7% ± 31.5%) was identified for individual MOS 3W that was stably maintained at all body sites, but remained low in prevalence and abundance in all other individuals (Fig. 2). Three low-abundance signature taxa were also detected, including OTU_11 (within *Sporidiobolales*, 11.9% ± 14.7%) for individual TMB 3Y, OTU_23 (within *Basidiomycota*, 11.4% ± 9.2%) for individual FH 3Z, and OTU_20 (within *Malassezia*, 12.1% ± 13.1%) for individual QB 3Y.

### Individual-specific CRT contribute to intrapersonal temporal community dissimilarity.

**FIG 3:**
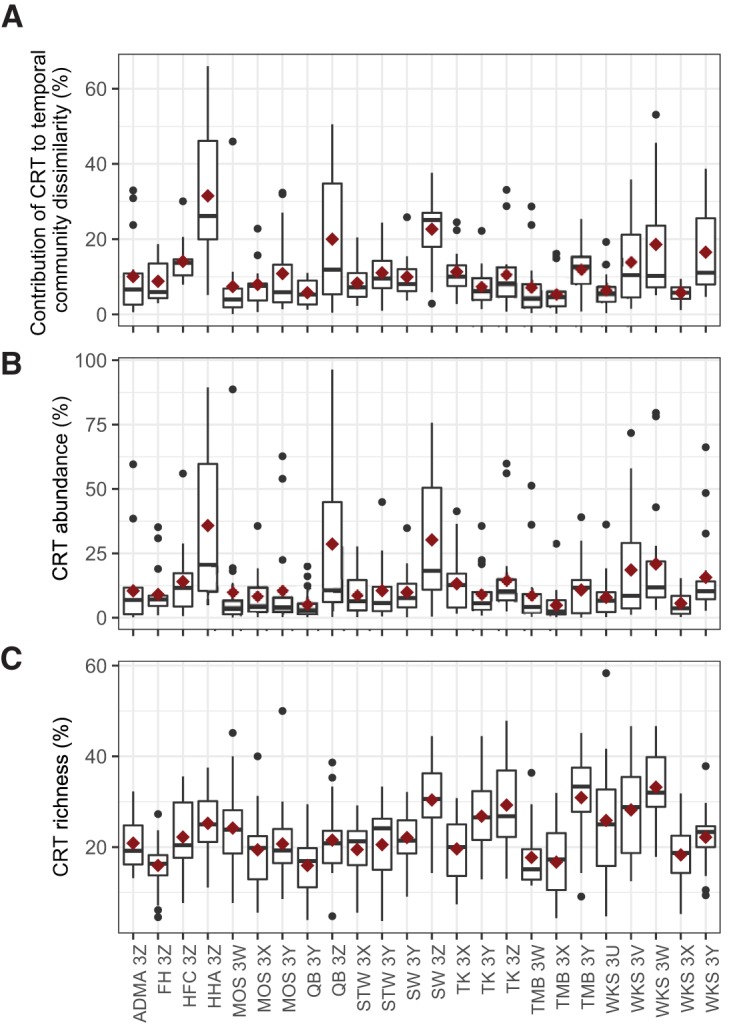
Contribution of individual-specific CRT to intrapersonal Bray-Curtis dissimilarity. (A) The contribution of individual-specific CRT to intrapersonal temporal community dissimilarity was measured at each body site of an individual between two successive seasons (in the order of winter-spring, spring-summer, and summer-autumn). (B and C) The abundance (B) and richness (C) of individual-specific CRT were calculated as the proportion of CRT counts or number in the total OTU counts or number of each sample in the rarefied individual-specific OTU tables, respectively. The crimson diamonds represent mean values.

In addition to the dominant taxa, between 32 and 82 CRT were identified for each host (see Fig. S3A in the supplemental material), of which most were non-human-associated taxa—e.g., plant pathogens and mushroom-forming fungi. Few CRT were unique to a single host (Fig. S3B), with cohabitants sharing more CRT than noncohabitants (Mann-Whitey *U* test, *P* = 0.04 [Fig. S3C]). Notably, a few CRT were exclusively detected in cohabitants, while being absent in individuals from other households. However, many CRT that were shared by cohabitants bloomed in a disjointed manner (Fig. S3D), suggesting that cohabitation might affect the presence of CRT in individuals or groups of cohabiting individuals, but not the timing of CRT blooms. Cohabitation is usually associated with similar exposome and lifestyle, which might contribute to the dynamics of CRT in high-exposure and high-perturbation skin communities. The contribution of CRT to intrapersonal temporal community dissimilarity ranged between 0 and 66% (Fig. 3A) and was positively correlated with CRT abundance or richness in each host community (Spearman’s rho = 0.96 and 0.58, *P* = 1.6 × 10^−6^ and 4.0 × 10^−3^ for CRT abundance and richness, respectively [Fig. 3B and C]).

10.1128/mSystems.00004-19.3 FIG S3 Identification of individual-specific CRT. (A) The number of CRT identified for each host, with taxonomy assigned at the genus rank. (B) The percentage of CRT classified to the following three categories for each host: unique to a host, uniquely shared between cohabitants from the same households, or presented in different individuals (cohabitants and noncohabitants) from at least two households. (C) The number of CRT shared between individuals pairwise. (D) The blooming pattern across seasons of CRT that were shared by the cohabiting members in household SW is shown as an example. The relative abundances of the shared CRT on different body sites within the same individual and season were averaged and standardized into a Z-score. OTU_5845 (indicated in red box) is the CRT uniquely identified in the cohabitants of household SW. Download FIG S3, PDF file, 0.5 MB. Copyright © 2019 Tong et al. 2019 Tong et al. This content is distributed under the terms of the Creative Commons Attribution 4.0 International license.

### Seasonal assembly of skin community is driven predominantly by neutral processes.

**FIG 4:**
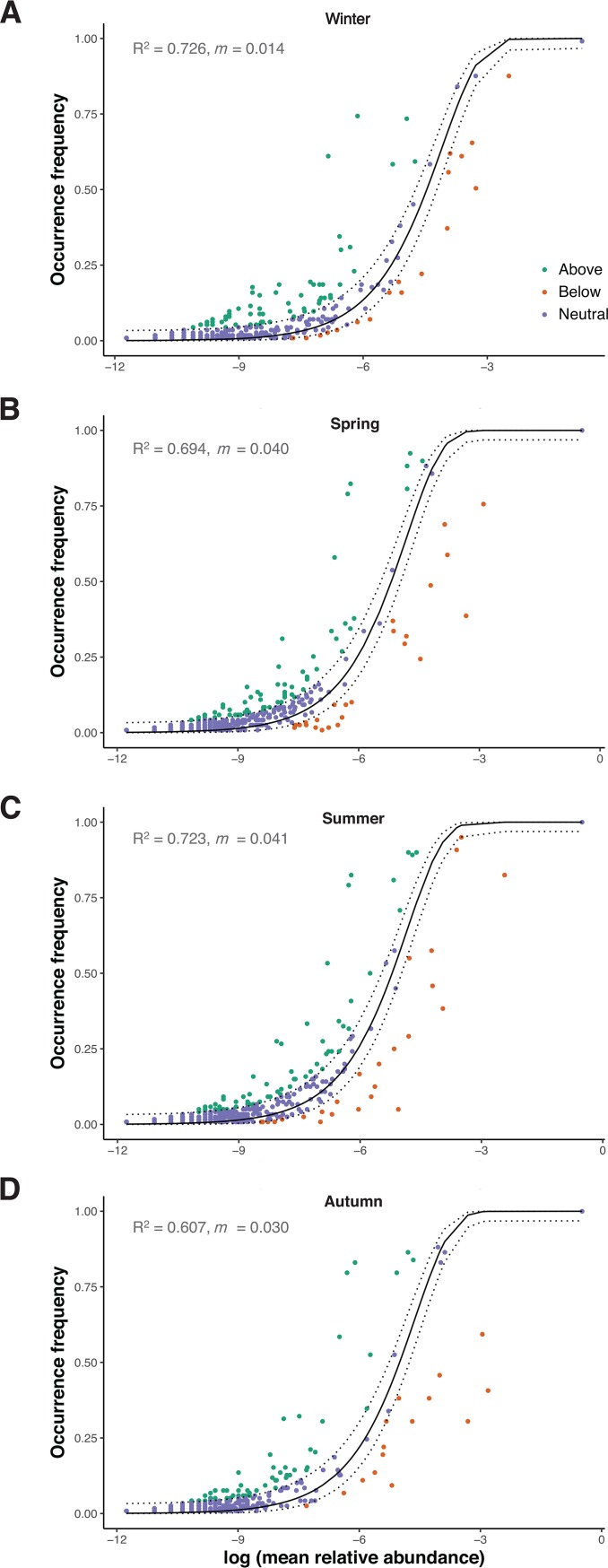
Fit of Sloan neutral model to skin mycobiomes. The occurrence frequency of OTUs was predicted for (A) winter, (B) spring, (C) summer, and (D) autumn skin communities according to the Sloan neutral model. OTUs that occurred more frequently than predicted by their abundance are colored in green, while those that occurred less frequently than predicted are shown in orange. Purple circles represent OTUs that are well fitted to the neutral model (i.e., within the 95% confidence interval). The predicted frequency is shown as a solid line, and dotted lines represent the 95% confidence intervals.

The Sloan neutral model (31) was fitted to the seasonal data set to investigate the contribution of stochastic processes to skin mycobiome assembly. Because this study spanned only 1 year, microbial speciation and diversification processes were unlikely to have a meaningful contribution to assembly and so were excluded from the model. The neutral model outperformed the binomial model for all seasons (see Fig. S4A in the supplemental material), suggesting that passive dispersal and ecological drift were more influential than random sampling of the metacommunity. The neutral model was well fitted to the skin communities for all seasons, with the *R*^2^ value ranging between 0.607 and 0.726 (Fig. 4). The estimated migration rate, *m*, a measure of the influence of dispersal on community composition, was lowest in winter and highest in summer, which might be associated with a preference for bare upper limbs in warmer weather. Within each season, 82 to 87% of OTUs were well predicted by the model (Fig. S4B). This finding suggests that neutrally distributed OTUs contributed a large proportion of skin community richness and thus that skin mycobiome assembly was predominantly driven by neutral processes.

10.1128/mSystems.00004-19.4 FIG S4 Neutral and nonneutral partitions of skin metacommunity. (A) Comparison of the fit of the neutral model and the binomial model based on the Akaike information criterion score. (B) Proportions of OTUs in each season that fell into the above-neutral, below-neutral, and neutral partitions. (C) PCoA of the three partitions in each season based on the binary Jaccard distance metric (presence/absence). (D) The relative proportion of *Malassezia* OTUs in the three partitions. Download FIG S4, PDF file, 1.7 MB. Copyright © 2019 Tong et al. 2019 Tong et al. This content is distributed under the terms of the Creative Commons Attribution 4.0 International license.

Taxa in the above-neutral, below-neutral, and neutral partitions formed three distinct clusters that differed significantly in the principal-coordinate analysis (PCoA) across all seasons (permutational multivariate analysis of variance [PERMANOVA], *P* = 0.002, *t *=* *3.2 [Fig. S4C]). The differences between clusters were driven by a small number of taxa that were specific to the nonneutral partitions. Partitions above the neutral prediction were strongly distinguished by OTUs from the genus *Penicillium* (Kruskal-Wallis test, *P* = 0.024), while below-neutral partitions were strongly distinguished by OTUs from the genus *Candida* (*P* = 0.024), as well as taxa from the classes *Basidiomycota* (*P* = 0.040), *Sporidiobolales* (*P* = 0.021), and *Tremellales* (*P* = 0.006). *Malassezia* species are thought to be well adapted to proliferation in skin ecosystem as core commensals (39–41). Based on this assumption, the proportion of OTUs classified as *Malassezia* was calculated for each partition. As expected, *Malassezia* OTUs made up a large proportion of the above-neutral partition, which was much higher than in the neutral and below-neutral partitions (Fig. S4D). This pattern was consistent across seasons, highlighting the role of selection in the seasonal assembly of skin mycobiome.

All CRT identified in individual mycobiomes fell into the neutral or below-neutral partitions (except one CRT from individual ADMA 3Z, which fell into the above-neutral partition [see Fig. S5 in the supplemental material]), and more CRT followed a neutral than nonneutral distribution within a host. These findings suggest that the dynamics of neutrally distributed CRT are largely regulated by stochastic processes (e.g., passive dispersal and ecological drift). The below-neutral CRT are likely to be dispersal limited most of the time, but these taxa could be driven to bloom due to environmental changes specific to the host or transient major dispersal events (e.g., the host comes in contact with a major reservoir of the taxon).

10.1128/mSystems.00004-19.5 FIG S5 Neutral model fit of individual-specific CRT. Three individuals from households ADMA, FH, and HFC are given as examples. Download FIG S5, PDF file, 1.9 MB. Copyright © 2019 Tong et al. 2019 Tong et al. This content is distributed under the terms of the Creative Commons Attribution 4.0 International license.

### Coassociation networks of skin fungi.

**FIG 5:**
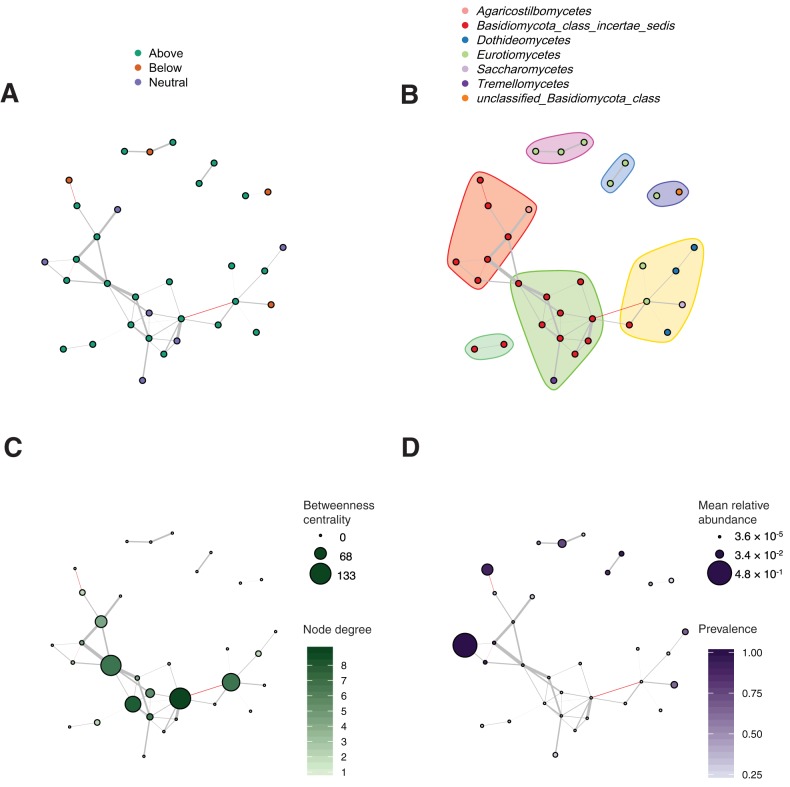
Coassociation networks of skin mycobiome in winter. Nodes in the networks represent OTUs, and edges are inferred associations between OTUs. Positive and negative associations are indicated by gray and red edges, respectively. Nodes without connections are excluded from the plots. (A) Nodes in the network are color-coded by the partition relative to the neutral model. (B) Nodes belonging to the same module are grouped by colored regions for visualization and color-coded by class lineages. (C) The intensity of the green is proportional to the node degree, and the node size is proportional to the centrality of the node in the network. (D) The intensity of purple is proportional to the prevalence of OTUs, and the node size is proportional to the mean relative abundance of the OTUs across all individual communities.

To infer potential ecological associations between fungal OTUs in the skin mycobiomes, coassociation networks were constructed on a seasonal basis using the Sparse InversE Covariance estimation for Ecological Association and Statistical Inference (SPIEC-EASI) framework (42). In each season, the average path length of the inferred network was compared to that of randomly assembled networks. The inferred networks were more highly interconnected than >96% of random networks with the same number of nodes and similar density, suggesting that the structure of the inferred networks is unlikely to be random (see Fig. S6 in the supplemental material). Interestingly, apparent network collapse was observed in autumn, with a lower network density, *D* (*D* = 0.0259), compared to those of winter (*D* = 0.0346), spring (*D* = 0.0311), and summer (*D* = 0.0299), suggesting that seasonality may affect the stability of the skin mycobiome network. In all seasons, nonneutral OTUs were overrepresented in the inferred networks, although this may be because of the frequency threshold applied (>25% prevalence), and they participated in more positive than negative coassociations in any season (Fig. 5A; see Fig. S7 in the supplemental material), suggesting that fungal OTUs tend to coexist or even develop mutualistic relationships in skin communities. In addition, the networks displayed a strong modularity (within a range of 0.5 to 0.62), with modules tending to be dominated by OTUs from a single taxonomic group (Fig. 5B). In each network, the OTU with the highest betweenness centrality was recognized as the hub node, which is likely the bottleneck (43) in the network. Surprisingly, all hub OTUs were very rare (abundance of <0.01% [see Table S1 in the supplemental material]), in contrast with the high-abundance OTUs, which tended to be less connected (Fig. 5C and D). This finding suggests that rare species might play important roles in stabilizing skin mycobiome communities over time.

10.1128/mSystems.00004-19.6 FIG S6 Average path length of inferred and random networks for each season. The dashed red line represents the average path length of the inferred network constructed using the SPIEC-EASI algorithm. Green bars represent the distribution of path length of the 10,000 random networks. Download FIG S6, PDF file, 1.5 MB. Copyright © 2019 Tong et al. 2019 Tong et al. This content is distributed under the terms of the Creative Commons Attribution 4.0 International license.

10.1128/mSystems.00004-19.7 FIG S7 Skin mycobiome coassociation networks in spring, summer, and autumn. Nodes in the networks represent OTUs, and edges are the inferred associations between OTUs. Positive and negative associations are indicated with gray and red edges, respectively. In all seasons, nodes without connections are excluded from the plots. (A, B, and C) Nodes in the network are color-coded by the partition relative to the neutral model prediction. (D, E, and F) Nodes belonging to the same module are grouped by colored regions for visualization purposes and color-coded by class lineages. (G, H, and I) The intensity of green is proportional to the node degree, and the node size is proportional to the betweenness centrality of the node in the network. (J, K, and L) The intensity of purple is proportional to the prevalence of OTUs, and the node size is proportional to the mean relative abundance of the OTUs across all individual communities. Download FIG S7, EPS file, 4.2 MB. Copyright © 2019 Tong et al. 2019 Tong et al. This content is distributed under the terms of the Creative Commons Attribution 4.0 International license.

10.1128/mSystems.00004-19.9 TABLE S1 Hub nodes in coassociation networks. Download Table S1, DOCX file, 0.02 MB. Copyright © 2019 Tong et al. 2019 Tong et al. This content is distributed under the terms of the Creative Commons Attribution 4.0 International license.

## DISCUSSION

From an ecological perspective, each human can be viewed as an island-like habitat patch (22), with a myriad of microorganisms colonizing inside and outside the body (12). As with any microbial community, an individual’s microbiome is assembled from a source pool (i.e., all the microbes a host encounters in its environment) via the fundamental processes of dispersal, diversification, selection, and drift (44). To understand the ecological processes driving the assembly of host-associated communities, neutral theory, which assumes species are functionally interchangeable, can act as a null model to contrast against deterministic effects such as niche specialization and selective pressure (33).

As the skin surface is highly exposed, the composition of the skin community is assumed to be susceptible to stochastic events such as randomly losing and acquiring microbes. Supporting this, this study found that the majority of the OTUs were well fit by the neutral model, suggesting that passive dispersal and ecological drift are more important than selection in skin community assembly. OTUs that deviate from the neutral model are likely to be under either host or other environmental selection pressure (positive/negative) or are able to disperse by specific routes not applicable to other OTUs in the source pool (32). Specifically, the skin-associated taxa that occurred more frequently than expected are likely to be well adapted to or actively selected by the hosts as moisture and nutrients on skin surface may benefit some microorganisms (1). On the other hand, environmentally associated taxa, which tended to be rarer, are more likely to be influenced by the diverse and dynamic nature of environmental exposures (45). Nevertheless, seasonality seems to play a less important role compared to selection forces in community composition of the below-neutral partition, given that a distinct cluster was identified on a PCoA plot. The existence of host-specific signature taxa is likely due to interactions between host-specific selective pressures, host-specific environmental exposures, and stochastic events.

Neutrally distributed taxa are less likely to be specifically adapted to a host. As a result, their abundances in any given community could be partly regulated by the surrounding source community via dispersal, independent of the niche functional traits (35, 36). Given the importance of neutral assembly processes, dispersal and drift have been suggested to be major drivers of the community variation both within and among hosts, as reported in the host-associated communities of fruit fly (35) and zebrafish (36). Within the same season, cohabitants harbored more similar communities than noncohabitants, consistent with the existence of a shared microbial source pool within a residence. For an individual host, the long-term community similarity was significantly lower than the short-term similarity, likely due to the fact that the shared source pool is itself dynamic and that neutral processes are powerful enough to generate a large amount of diversity even on short time scales (36).

Although neutrally distributed OTUs were mostly responsible for skin community richness, they were less likely to structure the coassociation network through interactions with other species. The prevalence of nonneutral OTUs in the coassociation networks suggests that nonneutral ecological processes may have a strong influence on network structure and composition, perhaps because healthy individuals tend to actively select for species that interact in ways that might benefit skin health (39, 46). The network analysis also found that hub OTUs were present in extremely low abundances, suggesting that rare species can have a disproportionate effect on community interactions (25). This suggests a possible answer to the question of why these low-abundance taxa do not drift to extinction (22) and implies that rare taxa that are stably present over long periods are very likely to be functionally significant. In addition, higher modularity in an association network has been interpreted as indicative of greater niche partitioning (47), with modules possibly representing functional niches (48). Thus, we hypothesize that these rare hub species might contribute to community function by bridging between niches. A recent study (49) has demonstrated that the extinction of rare species heavily influences the functional structure of species assemblages, but another report (50) has suggested that many rare species only add functional redundancy to an ecosystem. Further work is required to fully understand the functional roles of rare species in host-associated microbial communities. It is worth noting that fungi probably also interact with the skin bacterial community (51, 52), which was not considered in this study.

In summary, this study reveals considerable intra- and interindividual variations in skin fungal community composition over seasons. Most individuals cannot be distinguished from one another due to the absence of skin signature taxa, and individual-specific CRT only partly contribute to the temporal intrapersonal community dynamics. Neutrally distributed OTUs significantly contribute to skin community richness, and a large proportion of seasonal intra- and interindividual variations could be explained by neutral processes. However, neutral OTUs are less involved in community coassociation networks, while taxa that deviate above the neutral prediction are likely to interact with other OTUs and act as hub species to stabilize the network structure. In addition, seasonality seems to have an influence on the network stability of skin mycobiome, with a more fragile network in autumn than other seasons. Since the collapse of a network could be associated with potential cutaneous pathogens and skin diseases (4, 53), further epidemiological work is required to understand whether autumn is associated with increased risk of certain skin disorders and the links between skin fungal infection and weather conditions in Asian individuals. Overall, this study highlights the importance of neutral processes in the temporal dynamics and assembly of skin fungal communities and suggests that ecological models and network analysis can provide a useful framework to detect transient taxa and understand the health status of human skin microbiome.

## MATERIALS AND METHODS

### Sample collection and sequencing.

Subject recruitment, sample collection, genomic DNA extraction, library preparation, and sequencing were conducted as previously described (52, 54, 55). In brief, a total of 480 samples were swabbed from five skin sites (forehead, left and right outer forearms, and left and right palms) of 24 healthy occupants from 11 Hong Kong households across four seasons (in the order of winter, spring, summer and autumn) in 2014. These body sites were chosen to be representatives of sebaceous (forehead) and dry (palms and outer forearms) skin ecosystems. For each individual, a single biological sample was collected from each body site during a given season. Participants were instructed not to use makeup or skin care products at least 1 h before sampling. No antimicrobial medication was used at least 3 months prior to sampling. The subjects’ basic personal information and local weather conditions are summarized in Table S2 in the supplemental material. A negative control was prepared using reagents from the DNA extraction kit and processed in parallel with the samples. Samples were placed randomly on 96-well plates, and PCR amplification was prepared in a UV-sterilized laminar flow hood. The first fungal internal transcribed spacer (ITS1) region was amplified with the primer set 18S*fw* (5′-GTAAAAGTCGTAACAAGGTTTC-3′) and 5.8S*rv* (5′-GTTCAAAGAYTCGATGATTCAC-3′) (10). Libraries were sequenced on an Illumina MiSeq platform (SeqMatic, Fremont, CA) to generate 250-bp paired-end reads.

10.1128/mSystems.00004-19.10 TABLE S2 Metadata of subjects and local weather conditions. Download Table S2, XLSX file, 0.01 MB. Copyright © 2019 Tong et al. 2019 Tong et al. This content is distributed under the terms of the Creative Commons Attribution 4.0 International license.

### OTU formation.

Raw forward and reverse reads were merged with the “-fastq_mergepairs” command in USEARCH (version 9.0.2132) (56) and trimmed to a uniform length of 273 bp with a maximum error rate of 0.5 error per read using the USEARCH “-fastq_filter” command. Sequences that passed quality filtering were demultiplexed and clustered into OTUs at 97% similarity following the UPARSE pipeline (57). OTU taxonomy was assigned based on a curated ITS database (10) using UCLUST in QIIME (version 1.9.1) (58). Chimeras were identified using the sensitive mode of the “uchime2_ref” command in USEARCH to maximize the detection sensitivity. OTUs classified to taxonomic lineages with an average relative abundance of greater than 3% in the negative control were considered contaminants. Chimeras, singletons, and contaminants were removed from the data set. After quality control, a total of 1,597 OTUs comprising 8,928,145 reads were retained for the downstream analyses.

### Alpha- and beta-diversity analyses.

Samples were rarefied to 1,086 reads per sample before community analysis, and 10 samples with fewer than 1,086 reads were discarded. The rarefaction depth was selected to minimize the loss of samples, with Good’s coverage estimator greater than 0.97, suggesting that community richness had been mostly captured. The abundance-based alpha-diversity metric Shannon index and the beta-diversity metric Bray-Curtis dissimilarity were calculated for the rarefied OTU table using the QIIME scripts “alpha_diversity.py” and “beta_diversity.py,” respectively.

The temporal stability of the skin community was assessed using the Bray-Curtis dissimilarity metric, with symmetrical body sites pooled. Between-sample dissimilarities were classified into three groups: within an individual, between cohabitants of the same household, or between individuals from different households. Each household had one to five occupants, and cohabitation is indicated by a common prefix (e.g., for individual TK 3Z, TK is the household name). Intervals between sample times were represented as an integer from 0 to 3, where 0 represents the same season, 1 represents winter to spring, spring to summer, and summer to autumn, 2 represents winter to summer and spring to autumn, and 3 represents three seasons apart starting from winter.

### *Malassezia* species-level identification.

As the curated ITS database was unable to provide taxonomic classification at the species rank, a custom species-level reference database with 90 ITS1 sequences for the common skin genus *Malassezia* was used as described previously (52). Briefly, reads belonging to *Malassezia* OTUs were filtered and classified against the reference database based on a sequence similarity of greater than 99% using the “-usearch_global” command in USEARCH. In the first round of classification, 29 out of 155 *Malassezia* OTUs were assigned to a known species or strain in the database. The remaining unclassified *Malassezia* OTUs were then interrogated against the NCBI nr database, and only those that matched reference sequences containing the complete ITS1 region with at least 99% identity were considered correct hits. With this approach, the majority of *Malassezia* reads (4.8 million out of 5.2 million) were classified to the species rank.

### Identification of CRT and the contribution of CRT to intrapersonal community dissimilarity.

CRT were identified for each individual (referred to as individual-specific CRT) using the R script described by Shade et al. (26). In brief, these transiently abundant taxa were identified from the rarefied individual OTU tables as OTUs with coefficient of bimodality greater than 0.9 and a peak abundance greater than 0.5% of the total community when blooming. This abundance threshold enables detection of taxa with subtle temporal changes, as a steep decline in the number of CRT was observed with an increasing abundance threshold (see Fig. S8 in the supplemental material). As Bray-Curtis dissimilarity is a scaled summation of the absolute difference in abundance between two communities, the contribution of CRT to intrapersonal community dissimilarity between two samples was calculated as a ratio with scaling summation with all taxa in the denominator and the summation attributed to CRT in the numerator.

10.1128/mSystems.00004-19.8 FIG S8 Influence of abundance threshold on the number of CRT detected. The *x* axis shows the minimum relative abundance of CRT when blooming. The default threshold of the algorithm is 0.5%. The *y* axis shows the number of CRT detected, which dramatically decreased with an increasing threshold. Variation in the number of CRT detected is small below a threshold of 4%. Download FIG S8, PDF file, 0.5 MB. Copyright © 2019 Tong et al. 2019 Tong et al. This content is distributed under the terms of the Creative Commons Attribution 4.0 International license.

### Identification of signature taxa using the random forest algorithm.

Random forest analysis was implemented using the package “randomForest” (59) (version 4.6-14) in R (version 3.3.0), with a rarefication depth of 1,086 reads per sample and 500 trees. This supervised machine-learning method was used to identify OTUs with high power to discriminate between individuals, by iteratively generating individual identification predictions and calculating the decrease in classification accuracy that would result from excluding a given OTU from the group of predictors. The algorithm was performed on a seasonal basis with all OTUs from a given season included in the analysis.

To identify signature taxa for each host community, the prevalence (i.e., proportion of samples in which a predictor is detected within an individual) as well as the mean relative abundance and the coefficient of variation (CV) of relative abundance across body sites and seasons were calculated for each predictor in each host. OTU predictors present at all body sites within a particular subject with a higher discriminatory power and low CV in relative abundance over time are defined as individual-specific signature taxa, as described by Oh et al. in a previous temporal study of human skin microbiota (11).

### Coassociation networks.

Skin community coassociation networks were constructed using the SPIEC-EASI (version 1.0.2) (42) software on a seasonal basis with all samples from a given season included in the analysis. The algorithm was executed in Meinshausen-Bühlmann neighborhood selection mode, with a minimum lambda of 0.01 and a subsampling number of 50. As the coassociation network could be biased by taxa that are confined to a specific individual, OTUs detected in <25% of the samples in each seasonal data set were excluded from the network analysis. The final network model was selected via the “stability approach to regularization selection” and was visualized using the R package “igraph” (version 1.2.2) (60) with unconnected nodes discarded. The modularity of the coassociation networks was calculated using the edge betweenness community detection method, and the node with the highest centrality was defined as the hub OTU. Network density (*D*) was calculated using the “edge_density” function of R package “igraph,” which is defined as the ratio between the number of edges and the number of all potential connections of a network.

To test the significance of coassociation network path lengths, 10,000 networks were randomly assembled with the same number of nodes and similar density as the original network for a given season. Average path length was calculated for the original and random networks using the “mean_distance” function of R package “igraph.”

### Seasonal assembly of skin community predicted by Sloan neutral model.

The Sloan neutral model (31) was applied to assess the importance of neutral process in the assembly of skin communities over seasons using the R code described by Burns et al. (36). In general, the model predicts that species that are more abundant in the metacommunity are more likely to disperse by chance, whereas less abundant species are more likely to go extinct due to ecological drift (31). For a given season, the frequency of occurrence of OTUs in a set of local communities (i.e., skin community of an individual host) and their mean relative abundances across the metacommunity (i.e., skin communities of all individuals) were fitted to the model. All OTUs in a season were sorted into the three partitions based on whether and how they deviated from the 95% confidence interval around the neutral model prediction: above-neutral (occurred more frequently and/or at greater abundance than predicted by the neutral model), below-neutral (occurred less frequently and/or at lower abundance than predicted), or neutral (within prediction). The 95% confidence interval was determined using the “Hmisc” (61) (version 4.1-1) package in R. The fit of the neutral model was compared to the fit of the binomial model based on the Akaike information criterion (AIC) scores. The binominal model was computed using the R code described by Burns et al. (36) as a build-in model. The goodness of fit of the neutral model to seasonal skin data was assessed using the coefficient of determination (*R*^2^), and the estimated migration rate was represented with the parameter *m*.

PCoA was applied to visualize the compositional difference between the three partitions across four seasons based on the binary Jaccard distance metric. Each partition in a season was treated as a distinct community and rarefied to an equal depth of 6,181 reads (total number of reads of the smallest partition).

### Statistical tests.

All statistical differences between groups used either the nonparametric Mann-Whitney *U* test (two groups) or Kruskal-Wallis test (three or more groups). Permutational multivariate analysis of variance (PERMANOVA) was conducted with the “adonis” function in R package “vegan” (62) (version 2.5-3). Spearman’s rank correlation coefficients were computed using the “cor.test” function in R.

### Ethics approval and consent to participate.

Ethics approval for subject sampling and publication of data originating from subjects included in this study was granted by the City University of Hong Kong Ethics Committee (reference no. 3-2-201312 [H000334]).

### Data availability.

Computer scripts are available at FigShare (https://figshare.com/s/4e41b1d0894aceb669f3). Raw sequencing reads (480 skin samples and one negative control) in this study have been deposited in the NCBI SRA archive under accession no. SRP126376.
