# Supplementary figures and images for: Neutral Processes Drive Seasonal Assembly of the Skin Mycobiome
Source: mSystems. 2019 Mar 26;4(2):e00004-19. doi: 10.1128/mSystems.00004-19 (PMC6435813; doi:10.1128/mSystems.00004-19)

**A**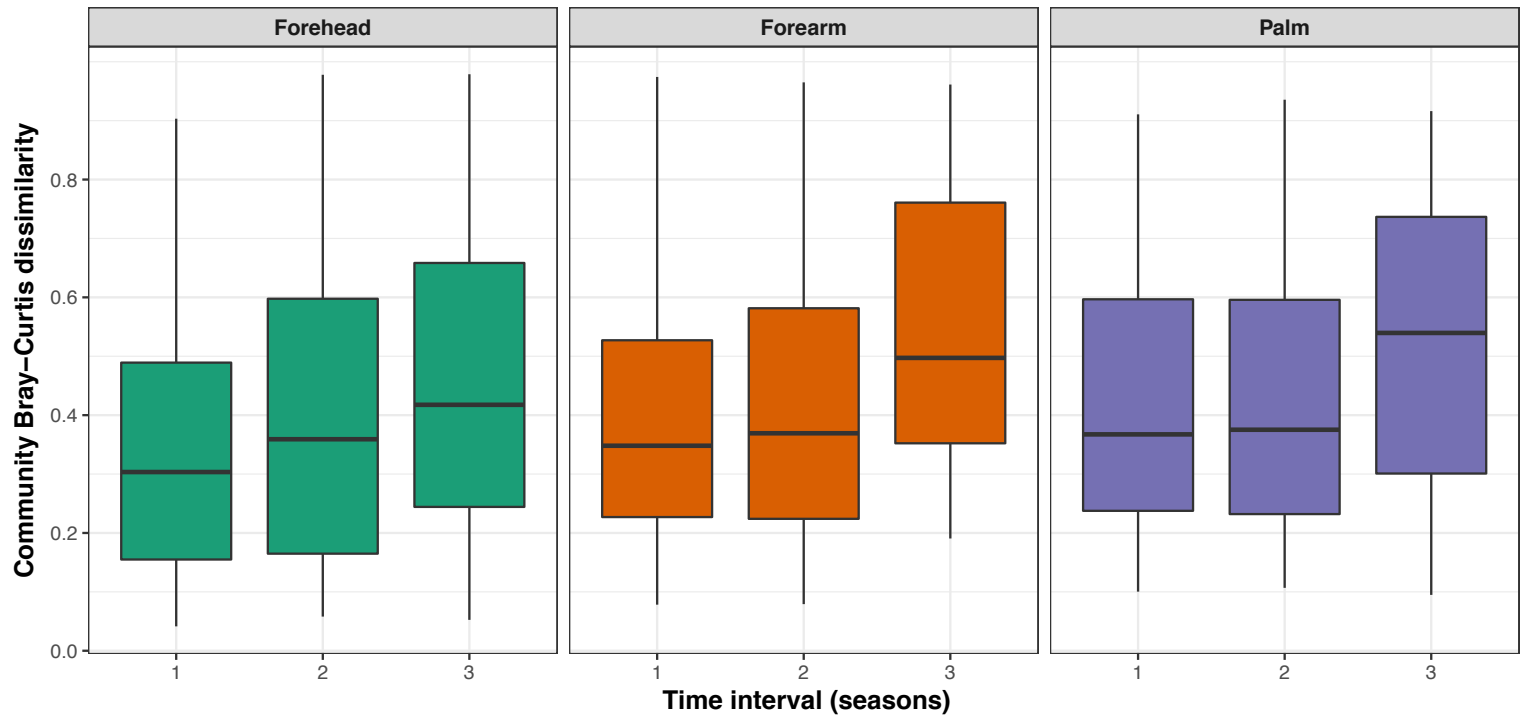**B**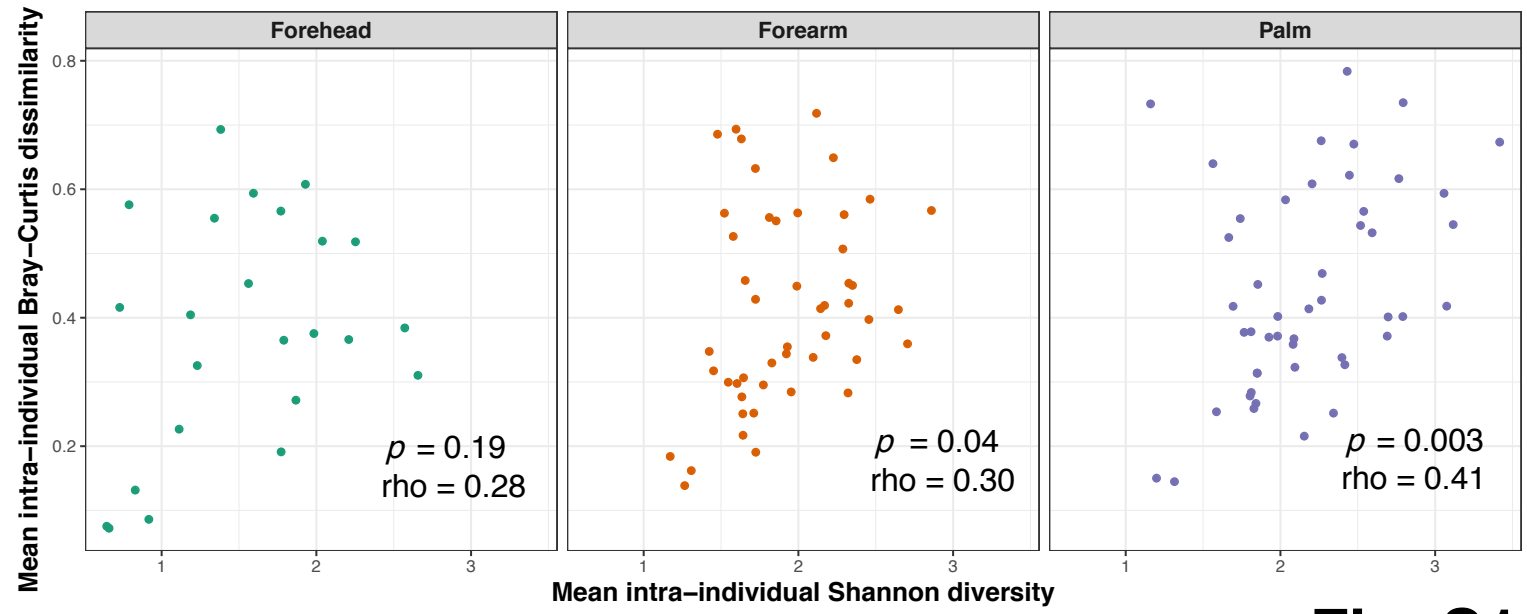**Fig. S1**

Supplement: FIG S1 [file mSystems.00004-19-sf001.pdf]

## Genus level Top 15 genera

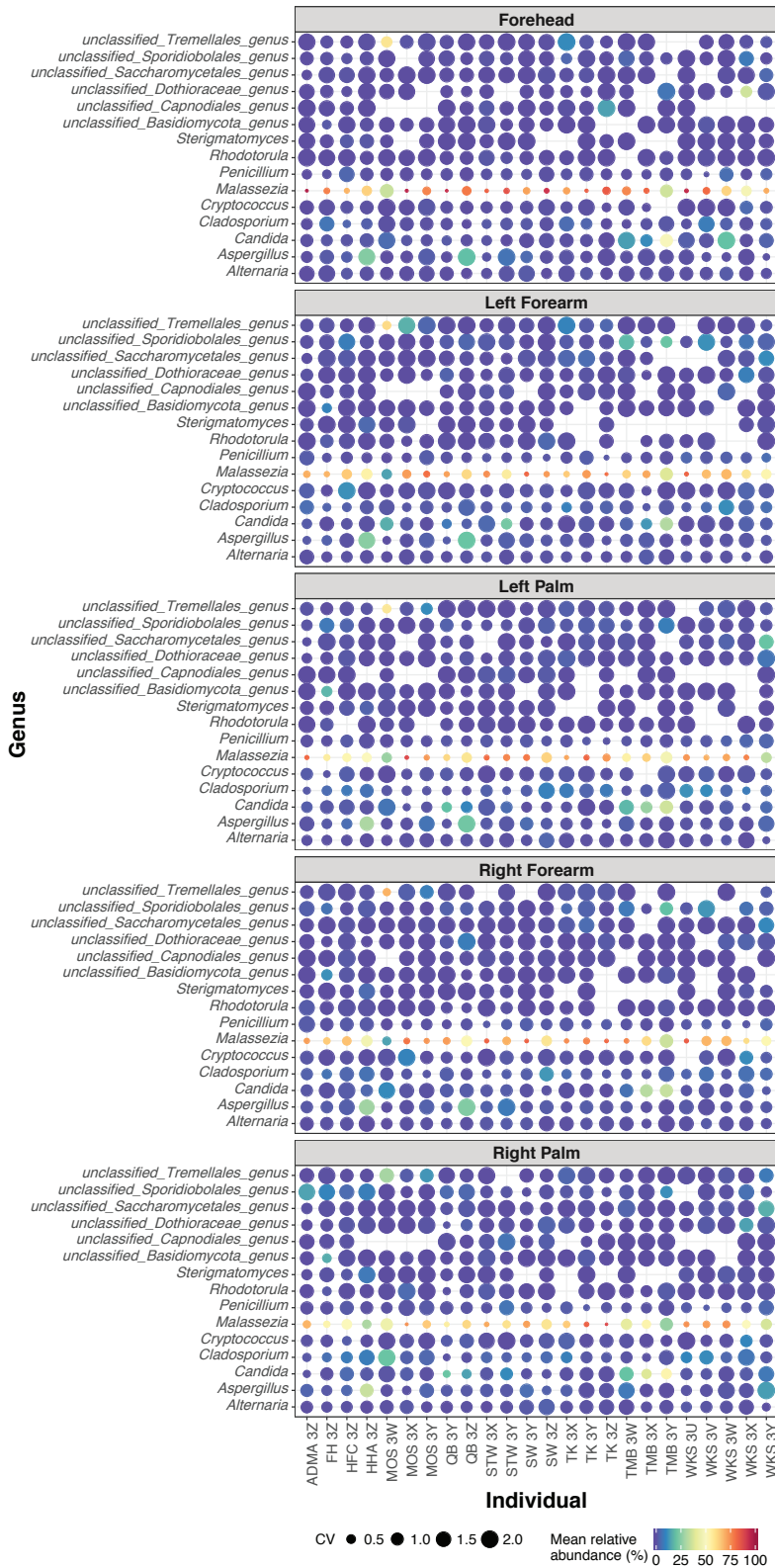

## Species level *Malassezia*

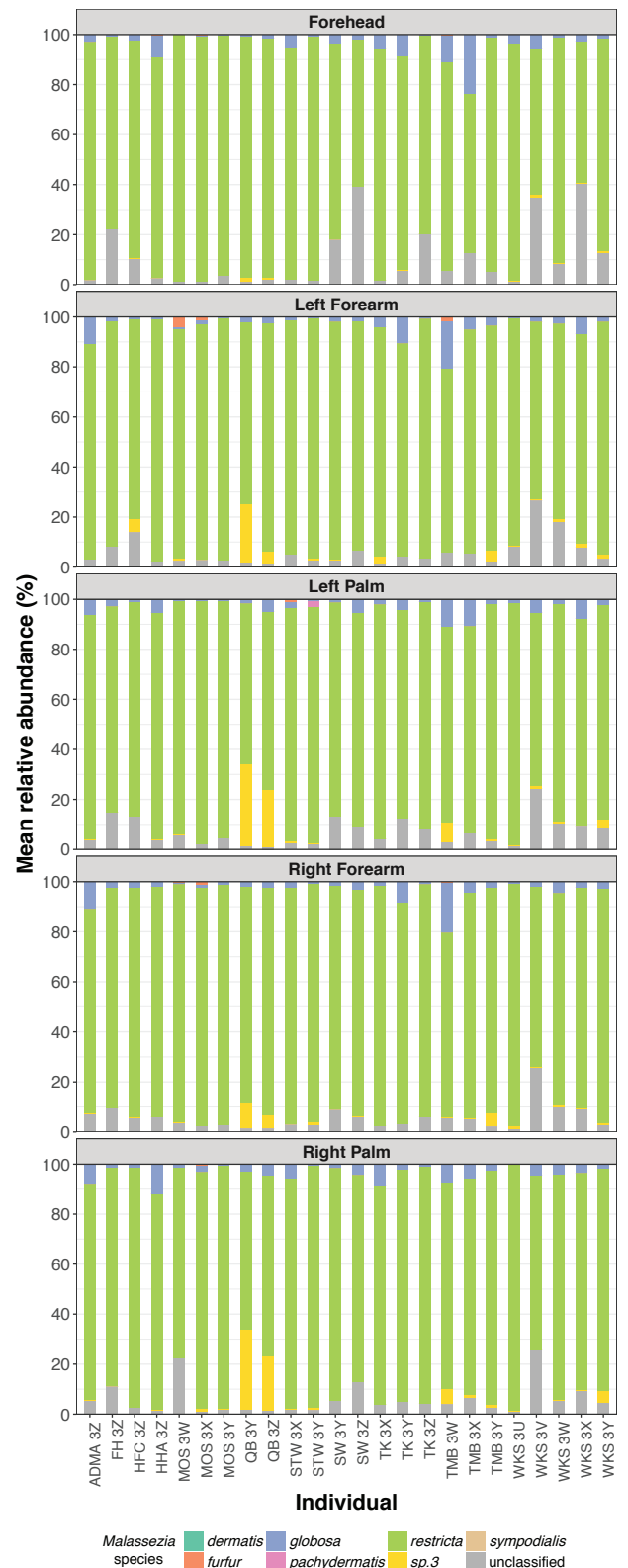

Fig. S2

Supplement: FIG S2 [file mSystems.00004-19-sf002.pdf]

A

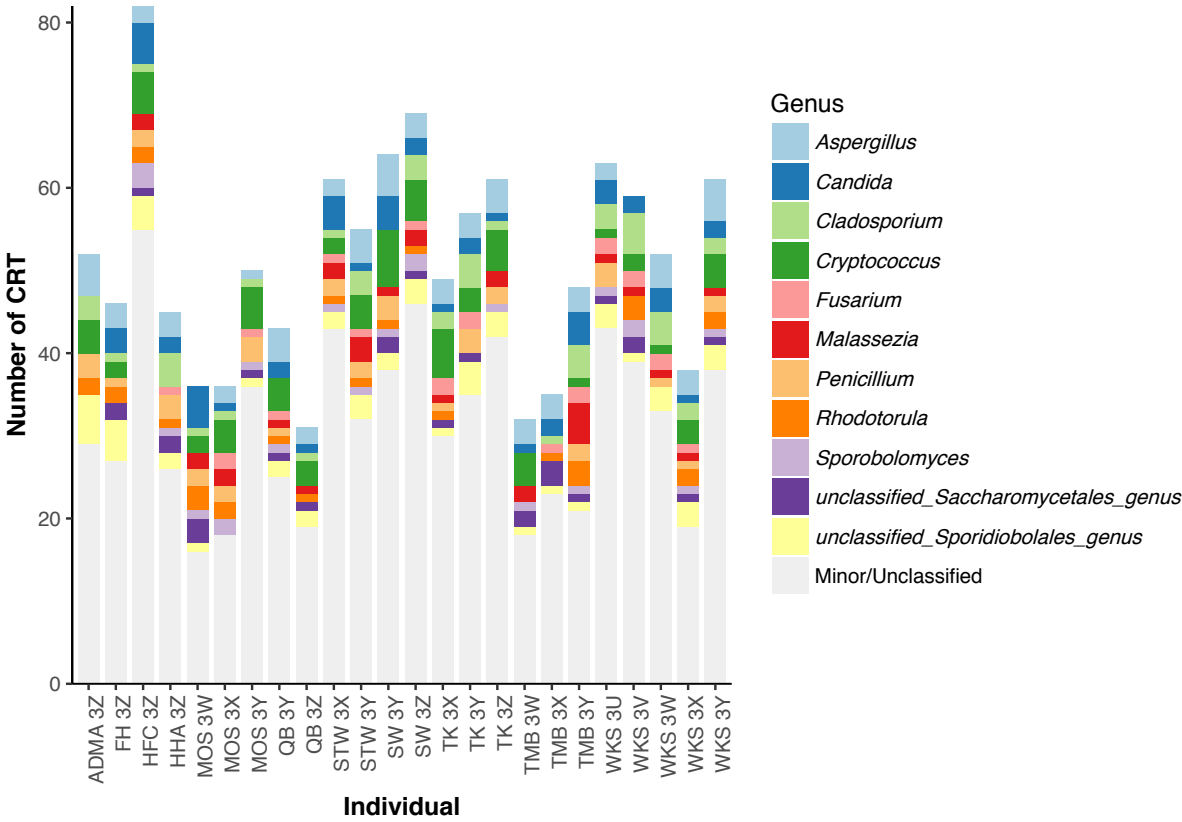

B

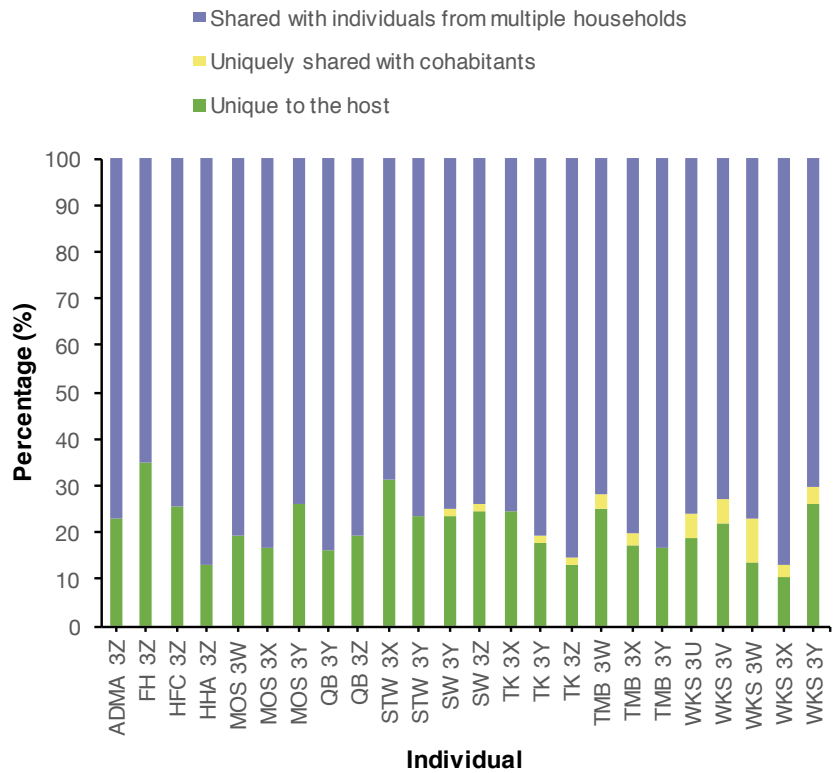

C

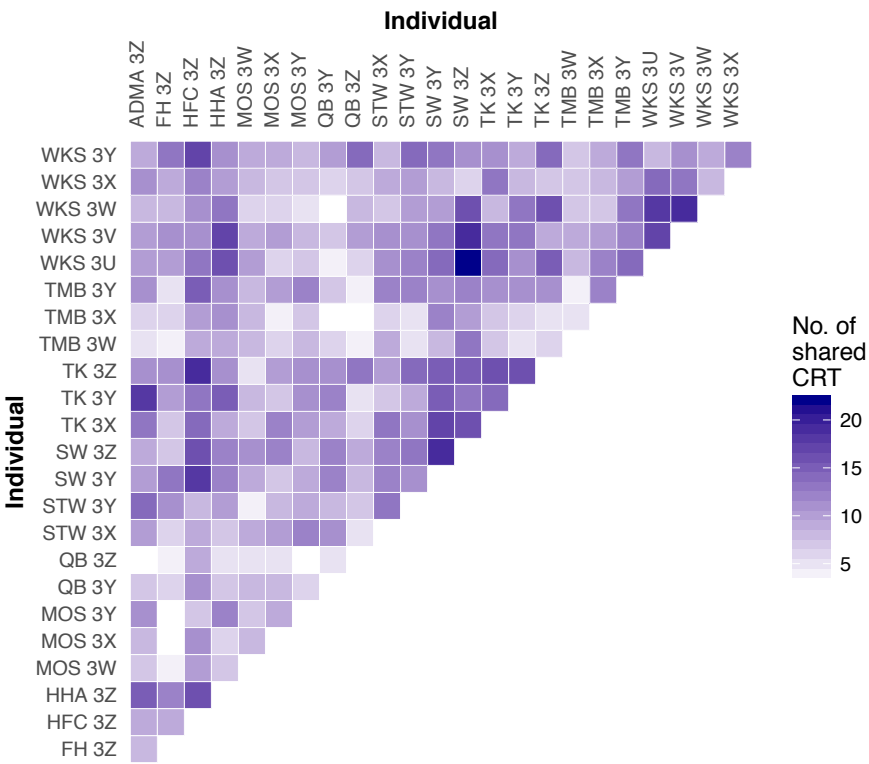

D

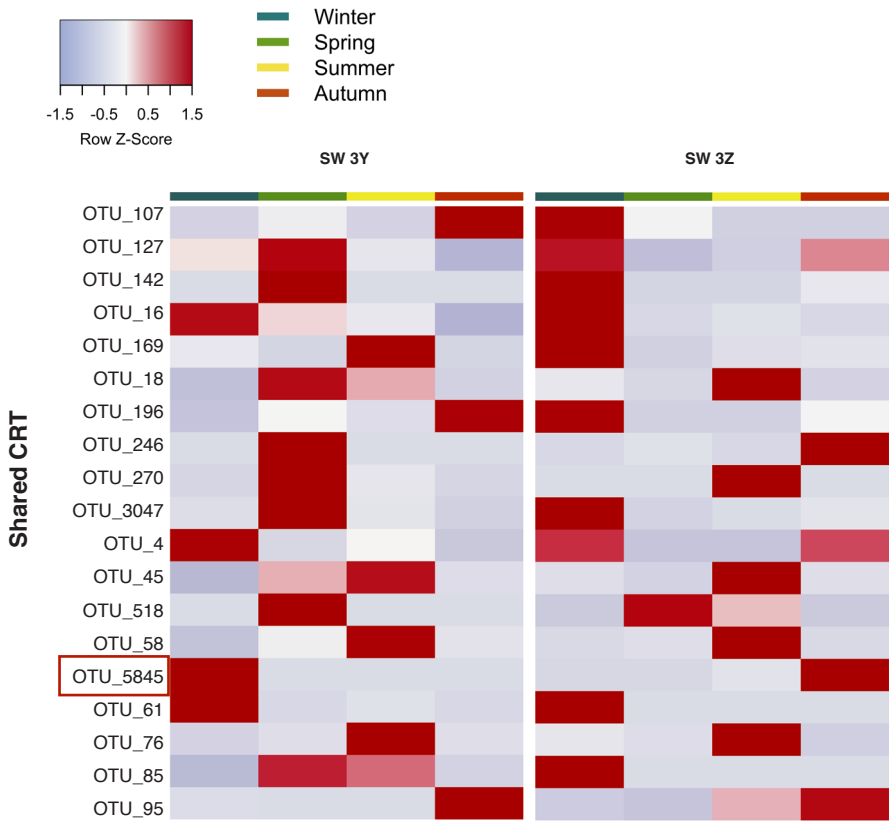

Fig. S3

Supplement: FIG S3 [file mSystems.00004-19-sf003.pdf]

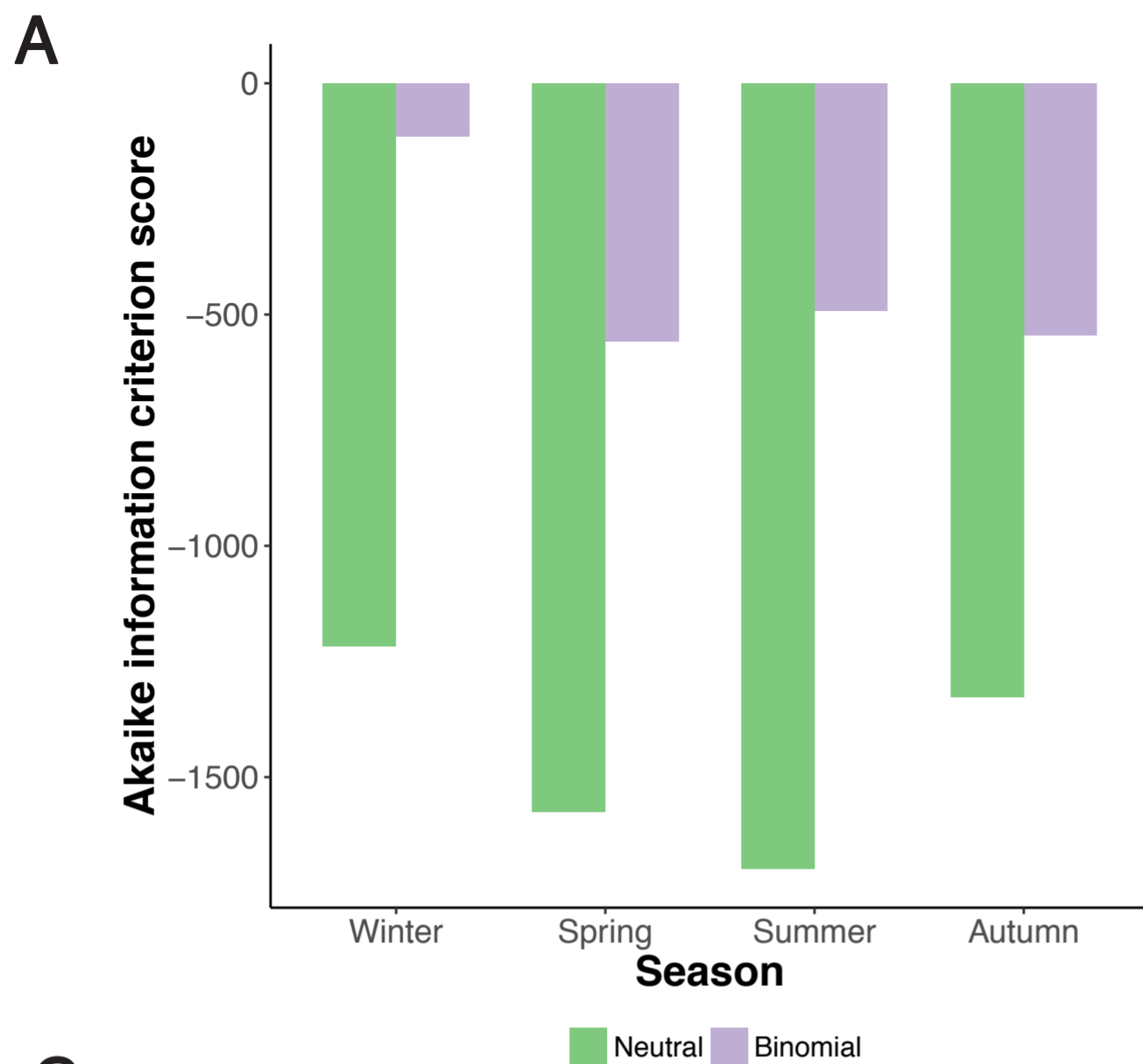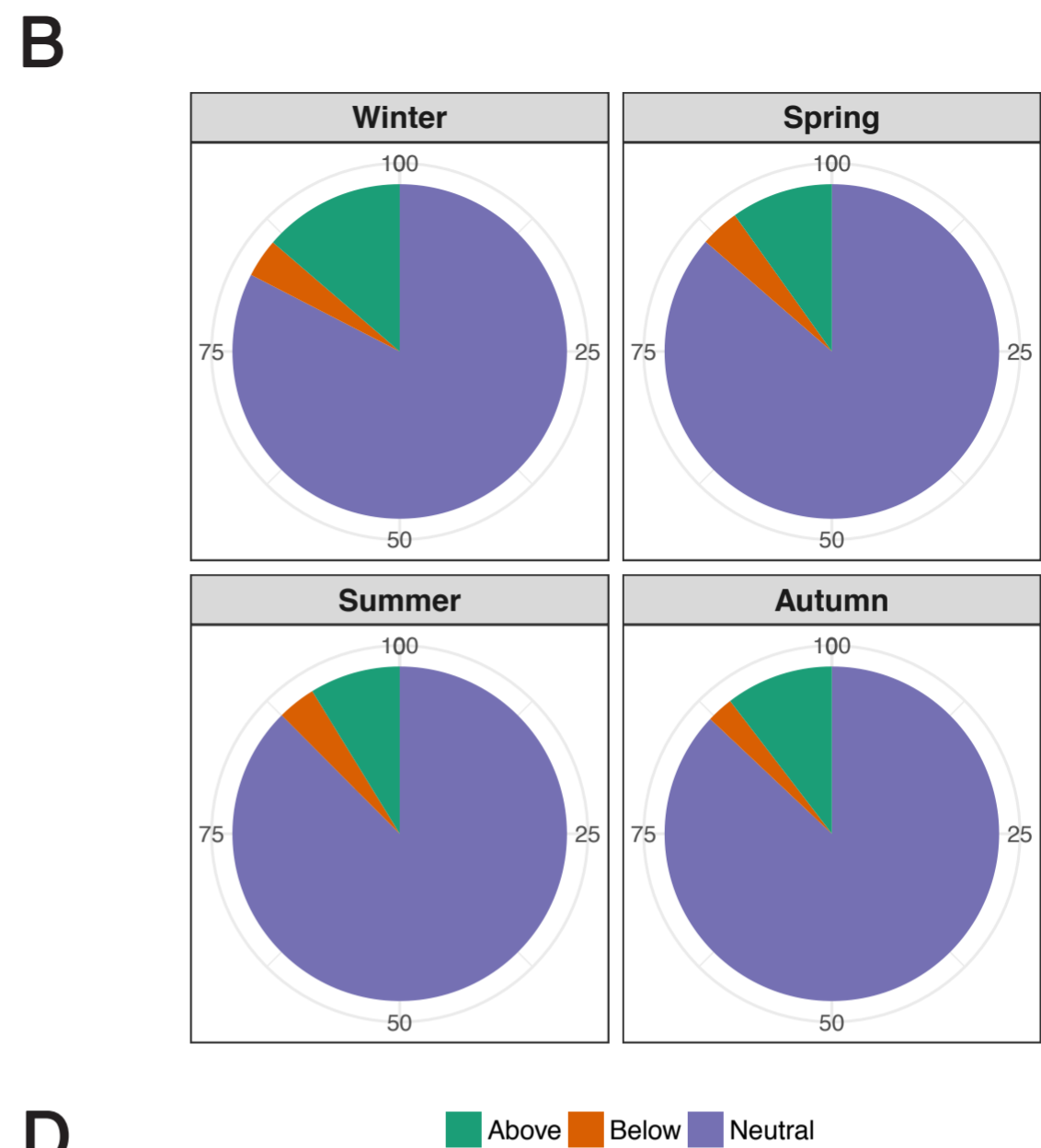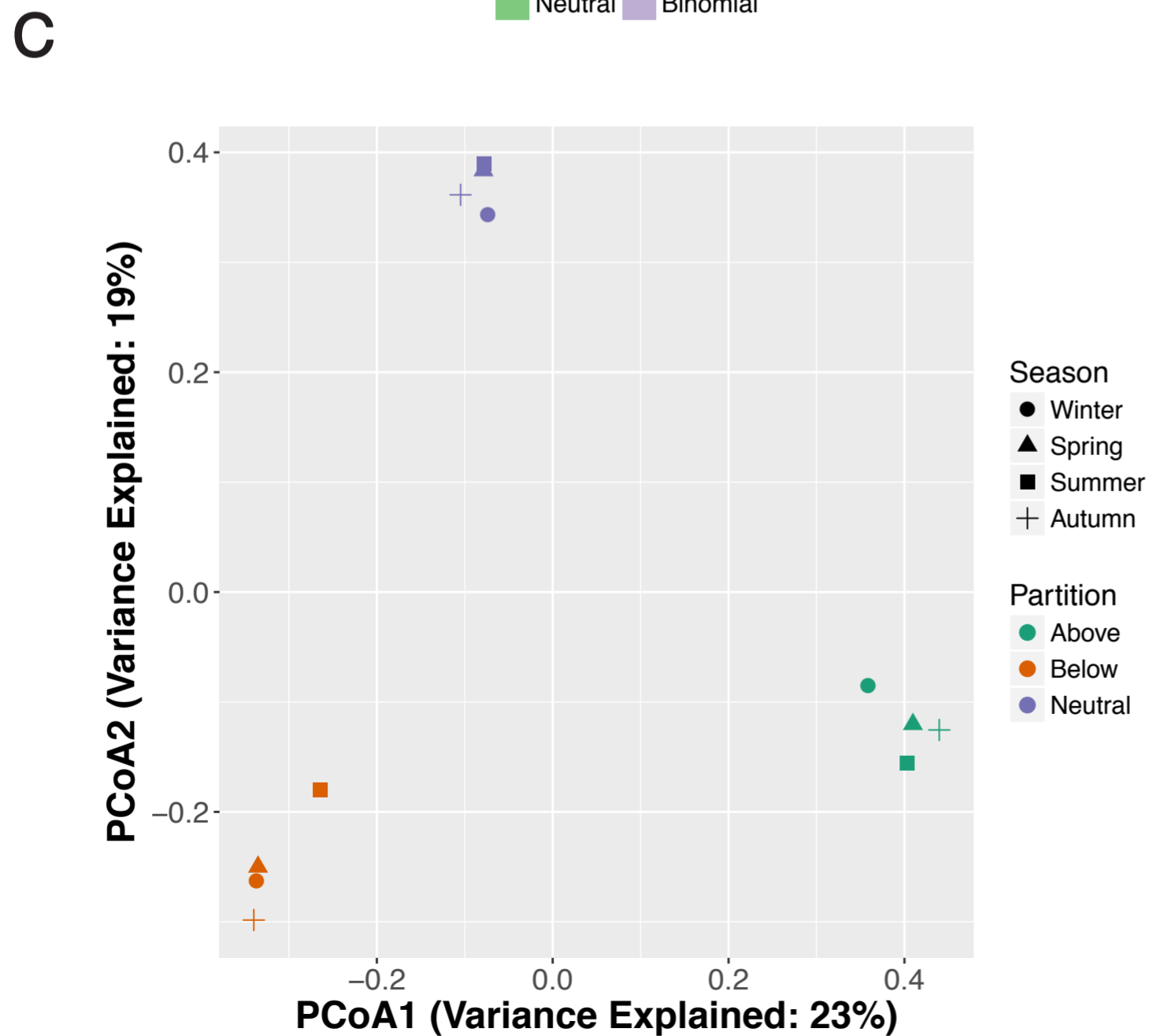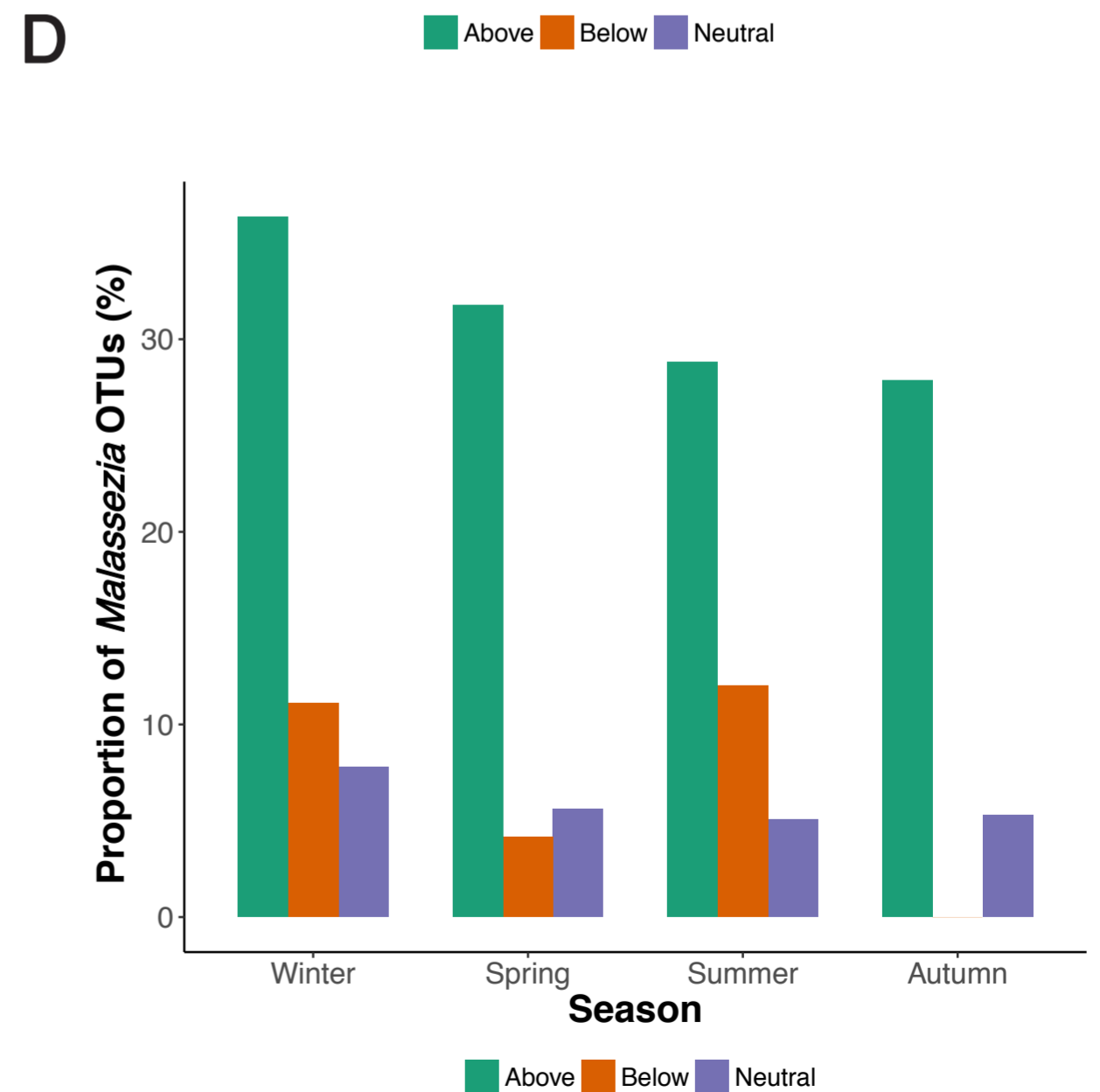

**Fig. S4**

Supplement: FIG S4 [file mSystems.00004-19-sf004.pdf]

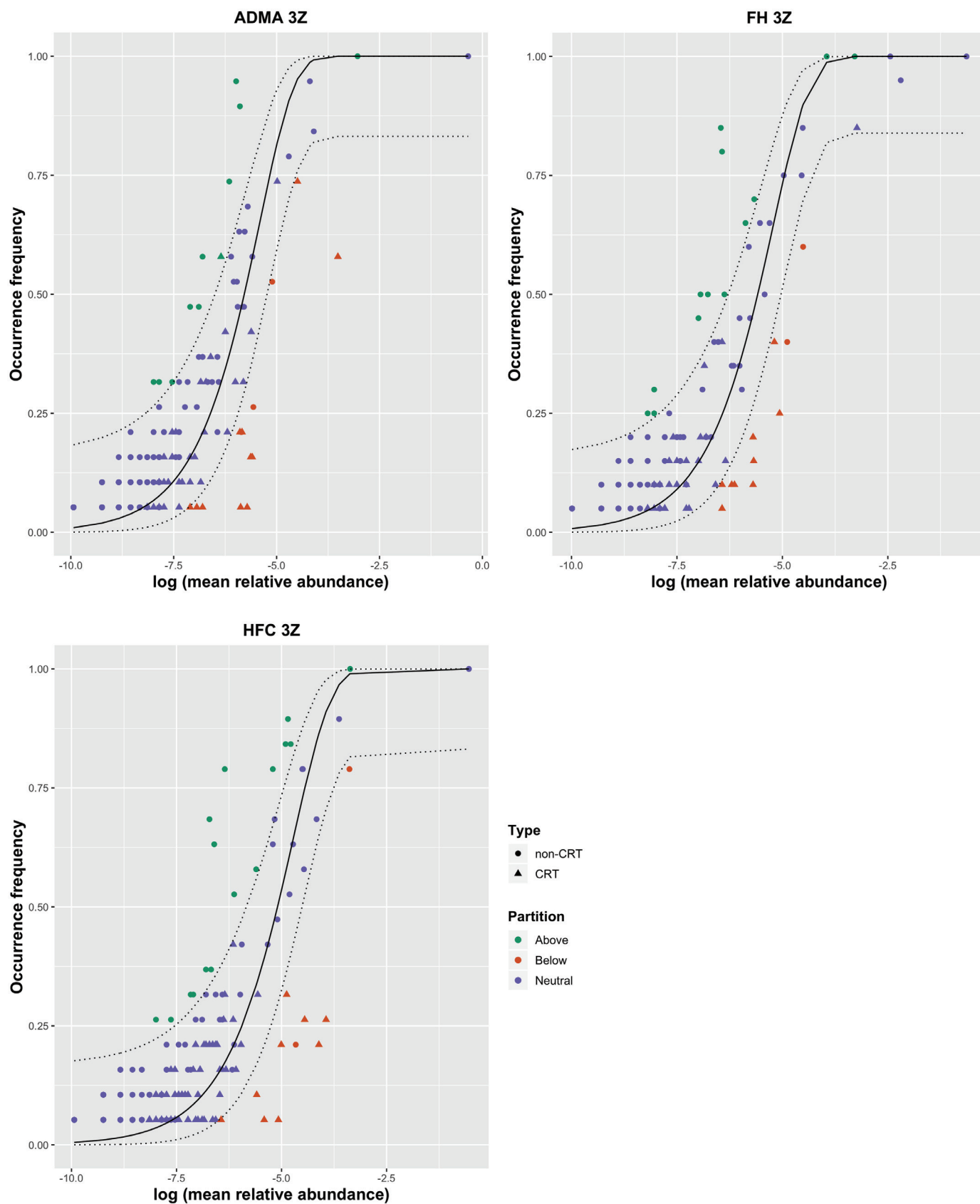

**Fig. S5**

Supplement: FIG S5 [file mSystems.00004-19-sf005.pdf]

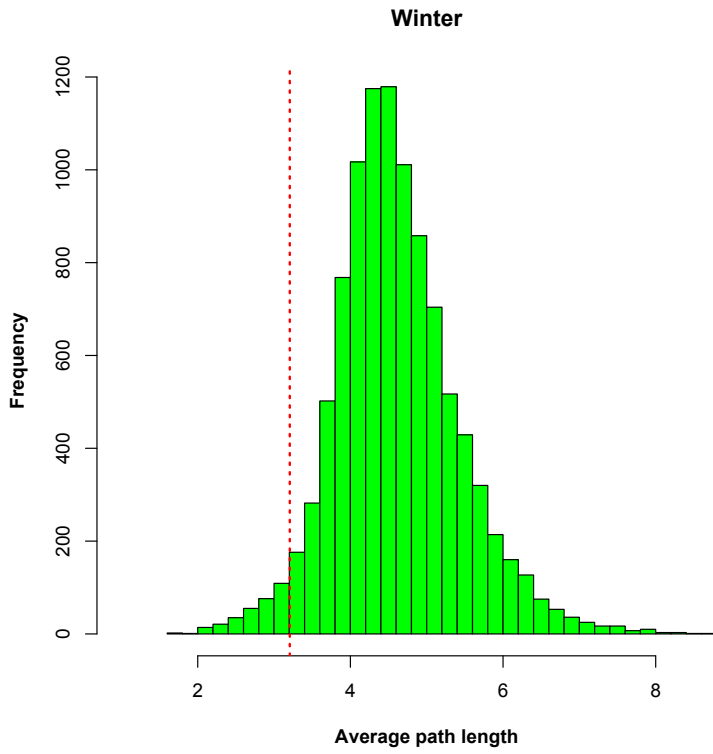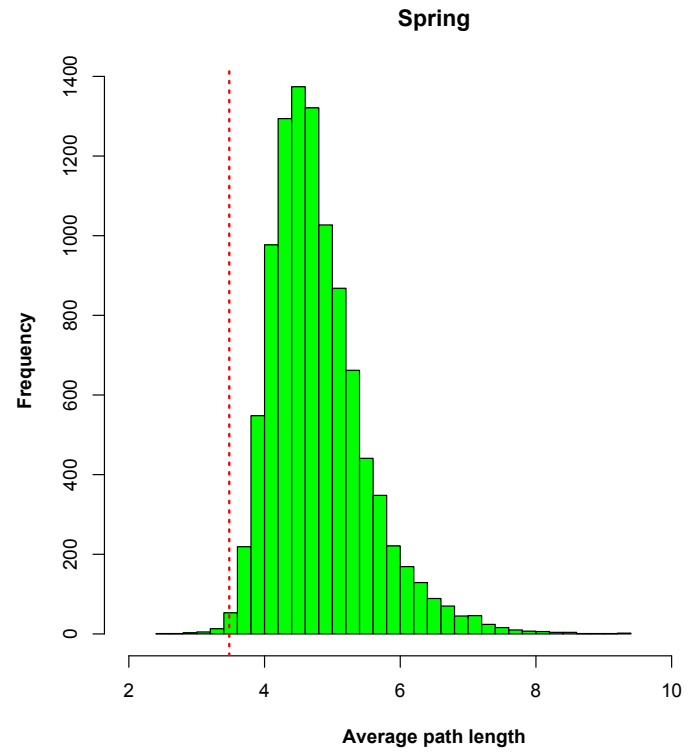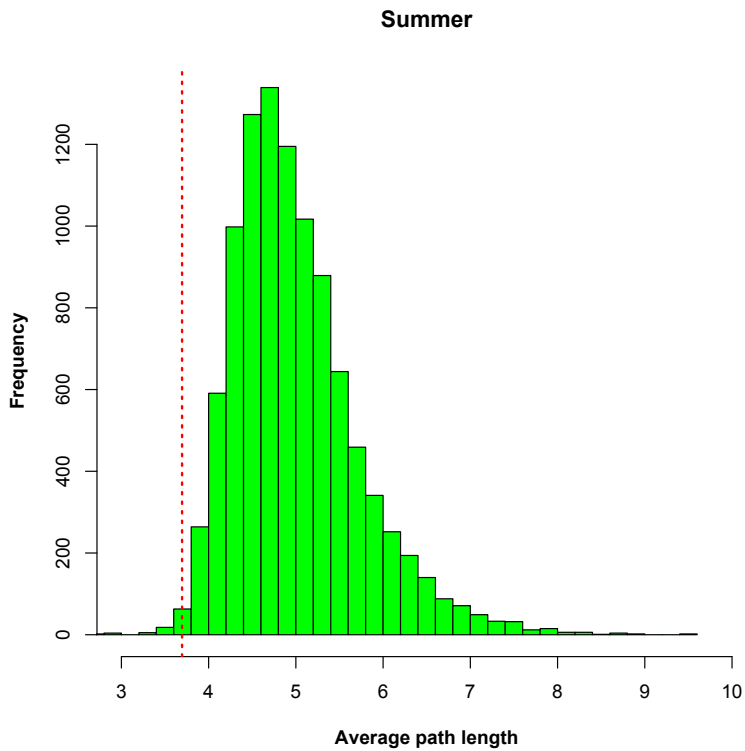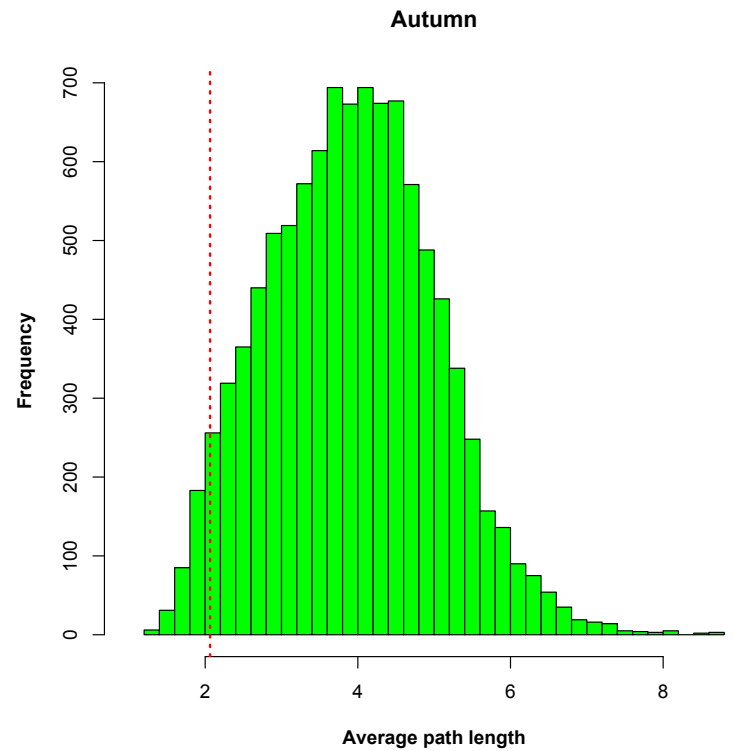

**Fig. S6**

Supplement: FIG S6 [file mSystems.00004-19-sf006.pdf]

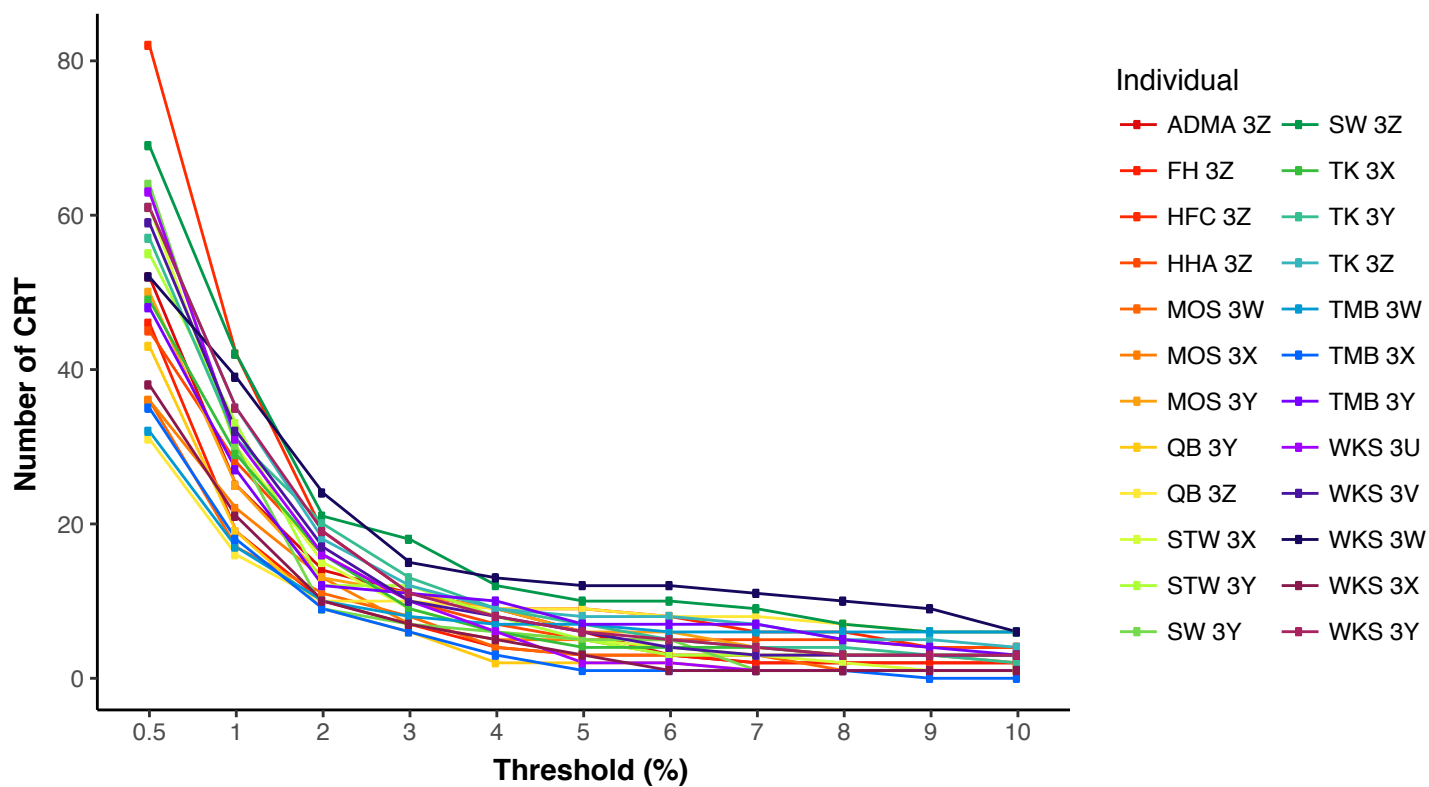

**Fig. S8**

Supplement: FIG S8 [file mSystems.00004-19-sf008.pdf]
