# Supplementary material for: Neutral Processes Drive Seasonal Assembly of the Skin Mycobiome
Source: mSystems. 2019 Mar 26;4(2):e00004-19. doi: 10.1128/mSystems.00004-19 (PMC6435813; doi:10.1128/mSystems.00004-19)
Supplement: TABLE S1 [file mSystems.00004-19-st001.docx]

| OTU | Season | Node Degree | Betweenness Centrality | Mean Relative Abundance (%) | Family | Genus |
| --- | --- | --- | --- | --- | --- | --- |
| OTU_11970 | Winter | 8 | 133.3 | 0.56 | *Malasseziales_family_incertae_sedis* | *Malassezia* |
| OTU_5848 | Spring | 8 | 190.2 | 0.29 | *Malasseziales_family_incertae_sedis* | *Malassezia* |
| OTU_10417 | Summer | 7 | 223.5 | 0.70 | *Capnodiales_family_incertae_sedis* |  |
| OTU_1384 | Autumn | 6 | 19.8 | 0.23 | *Malasseziales_family_incertae_sedis* | *Malassezia* |

**Table S1. Hub nodes in co-association networks.**
